# Alterations and Abnormal Mitosis of Wheat Chromosomes Induced by Wheat-Rye Monosomic Addition Lines

**DOI:** 10.1371/journal.pone.0070483

**Published:** 2013-07-30

**Authors:** Shulan Fu, Manyu Yang, Yunyan Fei, Feiquan Tan, Zhenglong Ren, Benju Yan, Huaiyu Zhang, Zongxiang Tang

**Affiliations:** State Key Laboratory of Plant Breeding and Genetics, Sichuan Agricultural University, Wenjiang, Chengdu, Sichuan, People’s Republic of China; University of Connecticut, Storrs, United States of America

## Abstract

**Background:**

Wheat-rye addition lines are an old topic. However, the alterations and abnormal mitotic behaviours of wheat chromosomes caused by wheat-rye monosomic addition lines are seldom reported.

**Methodology/Principal Findings:**

Octoploid triticale was derived from common wheat *T. aestivum* L. ‘Mianyang11’×rye *S. cereale* L. ‘Kustro’ and some progeny were obtained by the controlled backcrossing of triticale with ‘Mianyang11’ followed by self-fertilization. Genomic *in situ* hybridization (GISH) using rye genomic DNA and fluorescence *in situ* hybridization (FISH) using repetitive sequences pAs1 and pSc119.2 as probes were used to analyze the mitotic chromosomes of these progeny. Strong pSc119.2 FISH signals could be observed at the telomeric regions of 3DS arms in ‘Mianyang11’. However, the pSc119.2 FISH signals were disappeared from the selfed progeny of 4R monosomic addition line and the changed 3D chromosomes could be transmitted to next generation stably. In one of the selfed progeny of 7R monosomic addition line, one 2D chromosome was broken and three 4A chromosomes were observed. In the selfed progeny of 6R monosomic addition line, structural variation and abnormal mitotic behaviour of 3D chromosome were detected. Additionally, 1A and 4B chromosomes were eliminated from some of the progeny of 6R monosomic addition line.

**Conclusions/Significance:**

These results indicated that single rye chromosome added to wheat might cause alterations and abnormal mitotic behaviours of wheat chromosomes and it is possible that the stress caused by single alien chromosome might be one of the factors that induced karyotype alteration of wheat.

## Introduction

Wide hybridization is one of the stresses that might trigger reorganization of the parental genomes [1]. Newly synthesized allopolyploids of many species were developed through wide hybridization and genetic/epigenetic alterations of these allopolyploids have been widely investigated [2]–[5]. Unequal chromosome division at mitosis has also been reported in many wide hybrids [6]–[10].

Wide hybridization between wheat (*Triticum aestivum*) and rye (*Secale cereale*) is an important cytogenetic and breeding tool in wheat. The genomic changes of triticales (X *Triticosecale* Wittmack) are extensively studied [11]. Some newly synthesized triticale lines have been found to show rapid genomic and epigenomic changes [12]–[15]. Alterations of chromosomal structure of rye chromosomes in triticales have also been discovered [16]–[19]. Triticales have been used to produce wheat-rye chromosome addition, substitution and translocation lines. These wheat-rye chromosome addition, substitution and translocation lines are the source materials for development of more highly engineered introgression lines for wheat improvement. Alterations of rye telomeric/subtelomeric heterochromatin were observed in several sets of wheat-rye disomic addition lines and substitution lines [20]. Chromosome instability and genome rearrangements in wheat-rye disomic addition lines were also reported [21]–[22]. Almost all these previous studies focused on the disomic addition lines and the alterations of the structure of rye chromosomes. In addition, the previous studies on abnormal mitosis have mainly focused on the position of parental chromosomes in nucleus and the chromosome elimination at early generation of hybrids [7]–[9], [23]. An alternative strategy would be to study monosomic alien addition lines (MAALs). In present study, the alterations and abnormal mitotic behaviours of wheat chromosomes induced by wheat-rye 4R, 6R and 7R monosomic addition lines were observed and discussed.

## Results

### FISH Pattern of ‘Mianyang11’ Chromosomes

**Figure 1:**
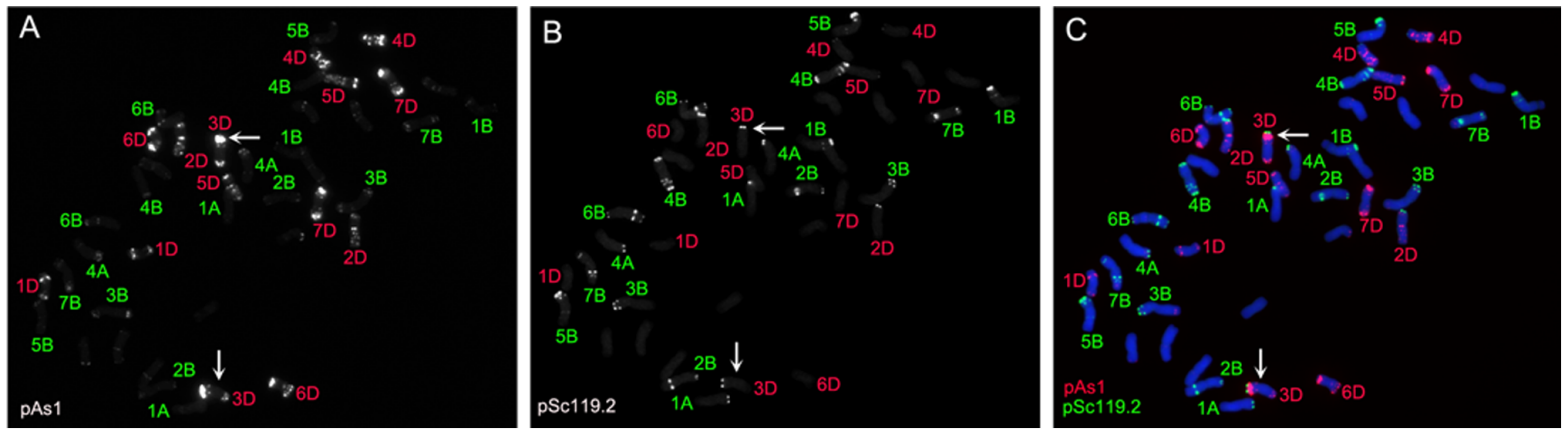
FISH was performed using pAs1 (red) and pSc119.2 (green) as probes on mitotic chromosomes of ‘Mianyang11’. **A** pAs1 FISH signals on chromosomes of ‘Mianyang11’, the DAPI-stained chromosomes were converted into a black and white image. **B** pSc119.2 FISH signals on chromosomes of the same cell in A, the DAPI-stained chromosomes were converted into a black and white image. **C** Merged image of A and B. Arrows indicate 3D chromosomes with pSc119.2 FISH signals. Chromosomes were counterstained with DAPI (blue).

The mitotic metaphase chromosomes of 20 ‘Mianyang11’ seedlings were analyzed by FISH using pAs1 and pSc119.2 as probes. pAs1 and pSc119.2 enabled the B- and D-genome chromosomes to be distinguished from each other, and also from chromosomes of the A-genome (Figure 1). The FISH patterns of pAs1 and pSc119.2 in all the mitotic cells of the 20 ‘Mianyang11’ seedlings were same. The pAs1 signals to D-genome chromosomes were agreement with Pedersen and Langridge [24] and the pSc119.2 signals to B-genome chromosomes were agreement with Sepsi et al. [25] The telomeric regions of 1AS, 4AL, 2DS, and 3DS apparently bore pSc119.2 signals (Figure 1B). The signals of pSc119.2 on 3DS were very strong (Figure 1B). In addition, the telomeric region of 4DS and 5DS bore weak pSc119.2 signals and the telomeric regions of 1D, 6D and 7D chromosomes did not bear pSc119.2 signals (Figure 1B).

### Structural Variation of 3D Chromosome Induced by 4R Monosomic Addition Line

**Figure 2:**
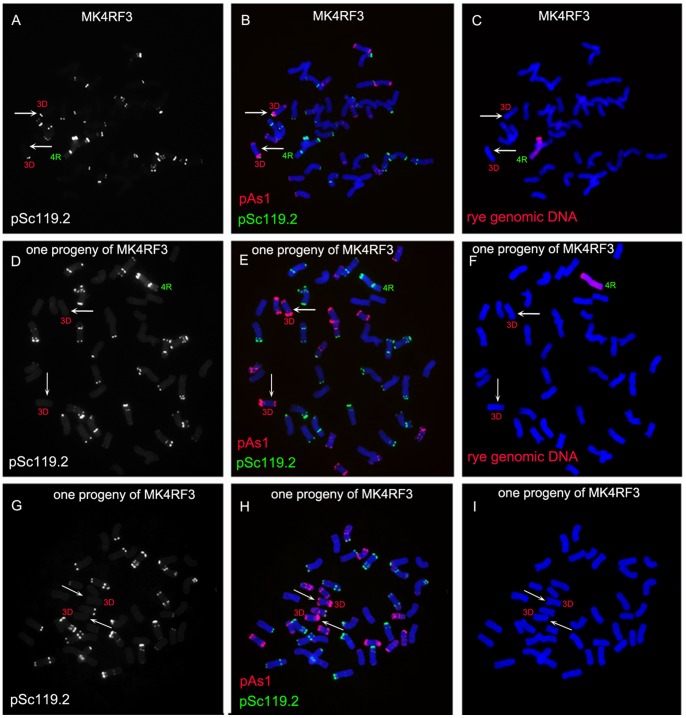
FISH and GISH using pAs1 (red), pSc119.2 (green) and rye genomic DNA (red) as probes on 4R monosomic addition line and its selfed progeny. **A** pSc119.2 FISH signals on chromosomes of MK4RF_3_, the DAPI-stained chromosomes were converted into a black and white image. **B** pSc119.2 and pAs1 FISH signals on the chromosomes of the same cell in A. **C** GISH analysis for the same cell in A and B. **D** pSc119.2 FISH signals on the chromosomes of one of the MK4RF_3_’s progeny that was 4R monosomic addition line, the DAPI-stained chromosomes were converted into a black and white image. **E** pSc119.2 and pAs1 FISH signals on the chromosomes of the same cell in D. **F** GISH analysis for the same cell in E and D. **G** pSc119.2 FISH signals on the chromosomes of one of the MK4RF_3_’s progeny that did not contain rye chromosome, the DAPI-stained chromosomes were converted into a black and white image. **H** pSc119.2 and pAs1 FISH signals on the chromosomes of the same cell in G. ** `** GISH analysis for the same cell in G and H and no GISH signals are observed. Arrows indicate the 3D chromosome with or without pSc119.2 FISH signals. Chromosomes were counterstained with DAPI (blue).

One wheat-rye 4R monosomic addition line was identified from the BC_2_F_3_ seeds by sequential FISH and GISH analyses. This 4R monosomic addition line was named MK4RF_3_. The strong signals of pSc119.2 could be observed on the telomeric region of the 3DS arms of MK4RF_3_ (Figure 2A–C). From the selfed progeny of MK4RF_3_, five wheat-rye 4R monosomic addition lines (BC_2_F_4_) were identified and the pSc119.2 signals could not be observed at the telomeric regions of 3DS arms of the five 4R monosomic addition lines (Figure 2D–F). Plants (BC_2_F_4_) that only contained 42 wheat chromosomes were also identified from the selfed progeny of MK4RF_3_, and the telomeric regions of the 3DS arms of these plants had no pSc119.2 signals (Figure 2G–I). The selfed progeny (BC_2_F_5_) of wheat lines that only contained 42 wheat chromosomes and the five wheat-rye 4R monosomic addition lines were subsequently analyzed by GISH and FISH and the pSc119.2 signals on 3DS arms were still not observed. This result confirmed the structural change in the 3DS telomeric region and indicated that the changed chromosome can be transmitted stably to the offspring.

### Alterations of 2D and 4A Chromosomes Induced by 7R Monosomic Addition Line

**Figure 3:**
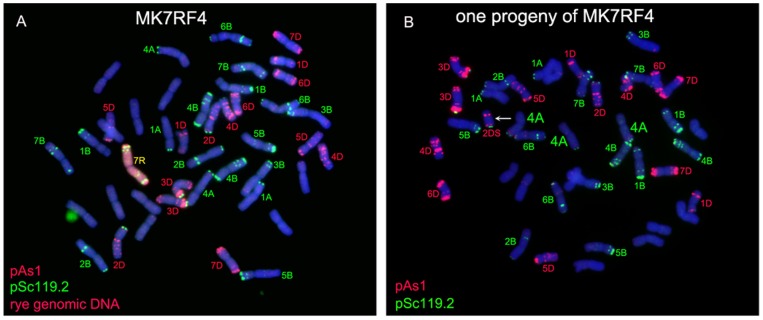
FISH and GISH using pAs1 (red), pSc119.2 (green) and rye genomic DNA (red) as probes on 7R monosomic addition line and its selfed progeny. **A** FISH and GISH analyses on the chromosomes of MK7RF_4_. **B** FISH and GISH analyses on the chromosomes of one of the selfed progeny of MK7RF_4_ and GISH signals can not be observed. The three 4A chromosomes are marked. Arrow indicates the broken 2D chromosome. Chromosomes were counterstained with DAPI (blue).

One wheat-rye 7R monosomic addition line was identified from the BC_2_F_4_ seeds by FISH and GISH analyses and this 7R monosomic addition line was called as MK7RF_4_. The normal structure of 2D chromosomes in MK7RF_4_ could be observed (Figure 3A), however, in one of the selfed progeny of MK7RF_4_, one 2D chromosome was broken and the 2DL arm was eliminated (Figure 3B). In addition, this wheat line contained three 4A chromosomes (Figure 3B). This result indicated that whea-rye 7R monosomic addition line induced the structural alteration of 2D chromosome and the variation of A-genome chromosomes.

### Mitotic Irregularities and Loss of 4B and 1A Chromosomes Induced by 6R Monosomic Addition Line

**Figure 4:**
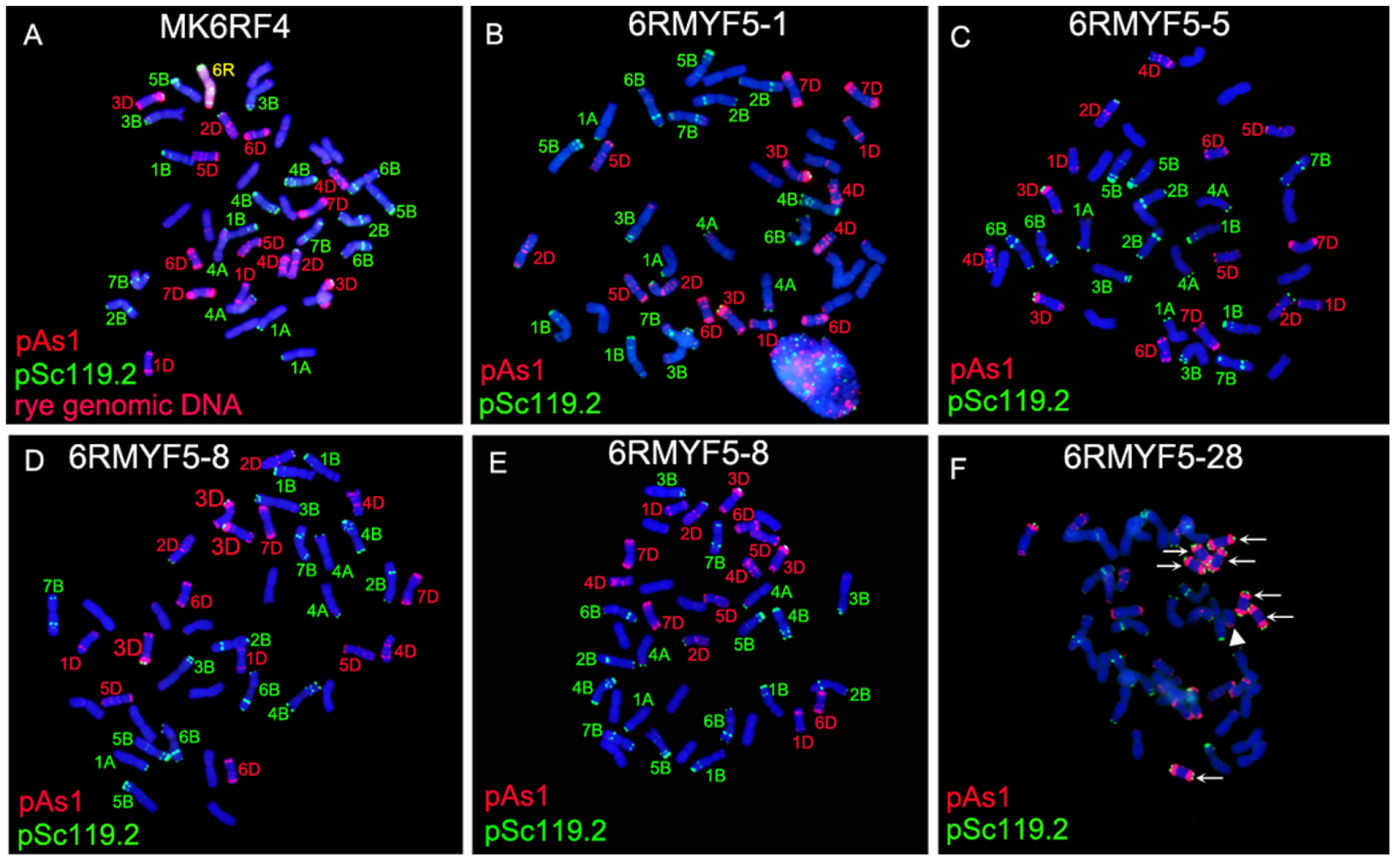
FISH and GISH using pAs1 (red), pSc119.2 (green) and rye genomic DNA (red) as probes on 6R monosomic addition line and its selfed progeny. **A** FISH and GISH analyses on the chromosomes of MK6RF_4_. **B** One 4B chromosome is eliminated in wheat line 6RMYF_5_-1. **C** Both of the 4B chromosomes are eliminated in wheat line 6RMYF_5_-5. **D** Three 3D chromosomes are observed and one 1A chromosome is eliminated in wheat line 6RMYF_5_-8. **E** One 1A chromosome is eliminated in wheat line 6RMYF_5_-8. **F** Seven 3DS isochromosomes and a broken D-genome chromosome are observed in wheat line 6RMYF_5_-28. Arrows indicate the 3DS isochromosomes. Triangle indicates the broken D-genome chromosome. No GISH signals were observed in B, C, D, E and F. Chromosomes were counterstained with DAPI (blue).

One wheat-rye 6R monosomic addition line was also identified from the BC_2_F_4_ seeds by FISH and GISH analyses and this 6R monosomic addition line was named MK6RF_4_. MK6RF_4_ had normal number of wheat chromosomes (Figure 4A). From the selfed progeny of MK6RF_4_, Seven noticeable lines 6RMYF_5_-1, 6RMYF_5_-3, 6RMYF_5_-5, 6RMYF_5_-8, 6RMYF_5_-11, 6RMYF_5_-15 and 6RMYF_5_-28 that did not contain rye chromosome were obtained. Wheat lines 6RMYF_5_-1, 6RMYF_5_-3 and 6RMYF_5_-11 contained 41 wheat chromosomes and one 4B chromosome was eliminated (Figure 4B). Wheat lines 6RMYF_5_-5 and 6RMYF_5_-15 only contained 40 wheat chromosomes and both of the 4B chromosomes were absent (Figure 4C). Sixty-one mitotic metaphase cells of line 6RMYF_5_-8 were randomly observed. The chromosome numbers in 20 of the 61 cells were 42 and the 20 cells contained three 3D chromosomes and lost one 1A chromosome (Figure 4D). The rest 41 cells contained two 3D chromosomes and one 1A chromosome was absent (Figure 4E). Fifty-eight mitotic metaphase cells of wheat line 6RMYF_5_-28 were randomly selected for observation. One of the 58 cells contained seven 3DS isochromosomes and a broken D-genome chromosome was observed (Figure 4F). Unfortunately, some of the wheat chromosomes in this cell could not be clearly identified because of indistinct mitotic spreads (Figure 4F). The rest 57 cells contained 40 chromosomes and 4B chromosomes were eliminated (data not shown). These results indicated that 6R monosomic addition line could induce the alterations of wheat chromosomes and abnormal behaviour of wheat chromosomes during mitosis.

## Discussion

### Alterations of Wheat Chromosomes in Wheat-rye Monosomic Addition Lines

Instability of rye chromosome in wheat-rye addition lines has been well documented. The deletions of 2RS arm, 3RL arm and 1AL arm were detected in wheat-rye disomic addition lines [26]. Among the set of Chinese Spring/Imperial disomic addition lines, only 4R and 6R chromosomes displayed no variation [20]. Bento et al. [22] discovered that chromosome structural rearrangements were more drastic in wheat-rye disomic addition lines than in triticale, indicating that the lesser the amount of rye genome introgressed into wheat the higher the likelihood of chromosome breakages occurring. In the present study, the structural variations of 3D and 2D chromosomes (Figure 2, 3B, 4F) and the loss of wheat chromosomes (Figure 4B–F) were apparently induced by wheat-rye monosomic addition lines. Ren et al. [27] reported that a single rye chromosome added to hexaploid wheat may cause the elimination of wheat chromosomes. The results in this study indicated that wheat-rye monosomic addition line could cause both elimination and structural variations of wheat chromosomes. Previous studies have already indicated that the single alien chromosome added to wheat genome was unstable [27]–[28]. From the present study, it should be noted that single alien chromosome added to wheat genome could also induce alterations of wheat chromosomes.

### Genetic Variations Induced by Monosomic Addition Lines

The disappearance of pSc119.2 signals from the telomeric regions of 3DS arms might be caused by the reduction of the copy number of this repetitive sequence. This case might occur during meiosis because the changed 3D chromosome could be observed in each of the detected root-tip cells. Further studies are needed to discover the mechanisms that caused the structural variation of 3DS arms. The changed 3D chromosomes were transmitted stably to next generation without any obvious additional structural changes. It is possible that the stress caused by single alien chromosome added to wheat might be one of the factors that induced karyotype alteration of wheat. It has already been reported that wheat-rye monosomic addition lines could induce different and drastic genetic/epigenetic variations of wheat [29]. In the present study, alteration of wheat chromosomes also embodied the genetic variations induced by monosomic addition lines. Therefore, alien monosomic addition lines should be one useful tool for chromosome engineering for the improvement of wheat cultivars.

### Abnormal Mitotic and Meiotic Behaviours Induced by Monosomic Addition Lines

In this study, one of the selfed progeny of MK7RF_4_ contained three 4A chromosomes and a broken 2D chromosome. Some progeny of MK6RF_4_ contained only one 4B chromosome or lost both of the 4B chromosomes. The changes of these wheat chromosomes should occur during meiosis because their alterations could be observed in each of the detected cells. That is, abnormal meiosis of 6R and 7R monosomic addition lines caused the phenomena that three 4A chromosomes existed in one cell, 2D chromosome was broken and 4B chromosomes were lost. However, some somatic cells of wheat line 6RMYF_5_-8 contained three 3D chromosomes (Figure 4D) apparently indicated the abnormal mitotic behaviour of 3D chromosome. In wheat line 6RMYF_5_-28, the seven 3DS isochromosomes in one cell (Figure 4F) should be formed during mitosis. If the 3DS isochromosomes were formed during meiosis, this kind of chromosomes could be seen in cell to cell. The 3DS isochromosomes in wheat line 6RMYF_5_-28 also displayed the structural variation of 3D chromosomes and their abnormal mitotic behaviour. Chromosomal bridge and micronuclei have already been observed in mitotic cells of triticale [17]. Unequal chromosome division occurred in somatic cells of wheat-rye allopolyploid has been reported [10]. Abnormal mitotic behaviour was also observed in the descendants of *T. aestivum* ×*Th. ponticum* amphiploid [30] and in common wheat carrying chromosome with gametocidal (Gc) chromosomes inducing breakage fusion bridge cycles in dividing root tip cells [31]-[32]. The chromosome fragmentation occurred in the 5-azacytidine treated root tip cells of 4S^L^ monosomic and disomic addition lines derived from *T. aestivum* Chinese Spring×*Aegilops sharonensis* was observed [33]. From these previous studies, abnormal mitotic behavious could be induced by allopolyploidization [10], [17], [30], Gc chromosome [31]–[32] and some exterior factors [33]. Obviously, the abnormal mitotic behaviours of wheat chromosomes in the present study had close relationship with 6R monosomic addition line. It was assumed that chromosome elimination in somatic cells of interspecific hybrids may be due to changes in the chromosome-spindle interaction and spindle abnormalities [6], [34]–[35]. Perhaps, the abnormal mitotic behaviour of 3D chromosome in wheat line 6RMYF_5_-28 was also caused by spindle abnormalities. It has already been reported that *Ph1* gene is a *trans-acting* gene affecting centromere-microtubules interaction [28]. Therefore, in order to discover the mechanisms that caused the abnormal mitotic and meiotic behaviours of chromosomes in wheat-rye monosomic addition lines, we should pay more attentions to the genetic and epigenetic variations of *Ph1* gene in monosomic addition lines.

In conclusion, to add a single rye chromosome to wheat genome could induce alterations and abnormal mitotic behaviours of wheat chromosomes. Further studies are needed to discover the mechanisms that wheat-rye monosomic addition lines cause alterations and abnormal mitotic behaviours of wheat chromosomes.

## Materials and Methods

### Plant Materials

Octoploid triticale line MK19-2 was obtained by crosses between common wheat *T. aestivum* L. ‘Mianyang11’ (genome AABBDD) and rye *S. cereale* L. ‘Kustro’ (genome RR). The parental wheat and rye plants were maintained by strict selfing. Some BC_2_F_3_, BC_2_F_4_ and BC_2_F_5_ seeds were obtained by the controlled backcrossing of MK19-2 with ‘Mianyang11’ followed by self-fertilization.

### Cytological Techniques and in situ Hybridization

FISH and GISH were used to analyze the mitotic metaphase cells of BC_2_F_3_, BC_2_F_4_, BC_2_F_5_ and control ‘Mianyang11’. The genomic DNA of rye ‘Kustro’, the *Aegilops tauschii* clone pAs1 and the rye clone pSc119.2 were used as probes. The genomic DNA of rye ‘Kustro’ and the repetitive sequences pAs1 were labeled with Texas Red-5-dUTP (Invitrogen). The repetitive sequences pSc119.2 was labeled with Alexa Fluor-488-5-dUTP (Invitrogen). The chromosome spreads of materials were prepared through the methods described by Han et al. [36]. Probe labeling and *in situ* hybridization were also operated according to Han et al. [36]. Images were taken using an epifluorescence Olympus BX51 microscope equipped with a cooled charge-coupled device camera operated with HCIMAGE Live software (version 2.0.1.5) and processed with photoshop CS 3.0. For each of the materials used in this study, two root tips were used and at least 40 cells were observed.
