# Correction: Alterations and Abnormal Mitosis of Wheat Chromosomes Induced by Wheat-Rye Monosomic Addition Lines

**DOI:** 10.1371/journal.pone.0106288

**Published:** 2014-08-18

**Authors:** The PLOS ONE Staff

The published article incorrectly refers to the wheat 1A chromosome throughout the article. The correct chromosome is the wheat 5A chromosome.
